# Generalized Epilepsy With Repetitive Sinus Pauses Following Generalized Tonic-Clonic Seizures Due to Reduced Baroreflex Sensitivity

**DOI:** 10.7759/cureus.39392

**Published:** 2023-05-23

**Authors:** Ryota Sasaki, Nahomi Osugi, Ichiro Nakagawa

**Affiliations:** 1 Department of Neurosurgery, Nara Medical University, Kashihara, JPN; 2 Department of Clinical Laboratory, National Hospital Organization Nara Medical Center, Nara, JPN

**Keywords:** sudden unexpected death in epilepsy, baroreflex sensitivity, cardioneuromodulation, electroencephalogram, syncope, generalized epilepsy

## Abstract

Epilepsy and syncope are sometimes difficult to differentiate, and they often occur together. We report here a unique case of severe neuromodulatory syncope associated with generalized epilepsy. A 24-year-old right-handed female with no remarkable history had her first epileptic seizure when she was 15 years old and was diagnosed with epilepsy. However, she had epileptic seizures or fainting spells every few months and was referred to Nara Medical Center at the age of 23 years. No obvious neurological abnormality was present, and no organic abnormality was found on head magnetic resonance imaging. The seizures were symmetrical generalized tonic-clonic seizures (GTCS) without aura, and the patient was unable to stand up for several hours after the seizure. Long-term video electroencephalogram monitoring revealed two types of seizures: (1) GTCS starting with generalized polyspikes and waves and (2) fainting with sinus arrest for up to 10 seconds when the patient tried to stand up after GTCS. After the addition of valproic acid following the diagnosis of generalized epilepsy, her epileptic seizures improved, but syncope remained. We consulted the cardiology department of our hospital and diagnosed mixed neuromodulatory syncope after performing the tilt test. She underwent catheter ablation for cardioneuromodulation, and her syncope improved. Several reports have described reduced baroreflex sensitivity during the interictal period in epilepsy, and seizure-related autonomic dysfunction has been implicated in sudden unexpected death in epilepsy (SUDEP). In addition to suppression of epileptic seizures, when autonomic nervous system symptoms associated with epilepsy are severe, as in this case, a thorough cardiovascular examination should be performed, and the patient should be treated with the goal of preventing SUDEP.

## Introduction

Loss of consciousness (LOC) is often encountered in daily practice and has a wide range of causes, including syncope, epileptic seizures, psychogenic non-epileptic seizures, and cerebrovascular accidents [1]. Syncope is defined as transient hypoperfusion of the entire brain and can occur when cerebral circulation is interrupted for six to eight seconds, systolic blood pressure drops to 60 mmHg, or oxygen delivery to the brain is reduced by 20% [2-4]. Differentiation between epilepsy and syncope is also important in epilepsy centers. In a prospective observational study in Italy, 45 (71.4%) of 63 patients with suspected epilepsy who had transient LOC without convulsions were reported to have syncope [5]. However, sometimes epilepsy and syncope occur together. We report here a case of generalized epilepsy with severe neuromodulatory syncope.

## Case presentation

**Figure 1:**
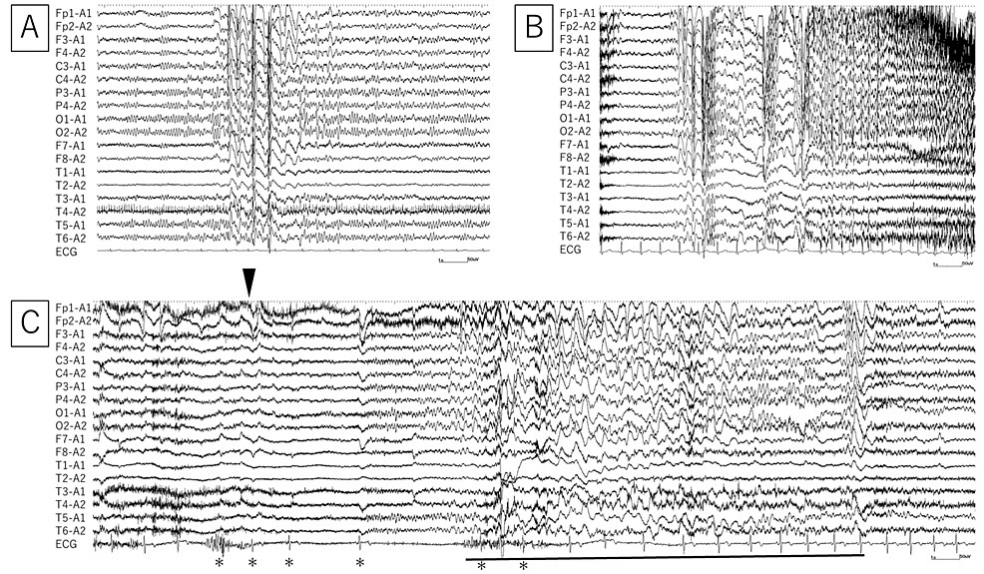
Interictal and ictal EEG findings. (A) Interictal EEG shows generalized spikes and wave complexes. (B) Ictal EEG shows GTCS onset with generalized fast waves following polyspikes and waves. (C) After GTCS, when the patient changes position from supine to sitting (arrowhead), the RR interval is prolonged on ECG (asterisk), and generalized slow waves follow (black line). Sampling rate: 500 Hz, high-frequency filter: 120 Hz, and time constant: 0.1 seconds. EEG: electroencephalogram; GTCS: generalized tonic-clonic seizure.

A 24-year-old right-handed female had a family history of febrile convulsions in her twin sister. Our patient was diagnosed with epilepsy at the age of 15 and started treatment with anti-epileptic drugs. However, due to LOC every few months, she was referred to Nara Medical Center at the age of 23. Her medications at the time of the first visit were levetiracetam 2000 mg/day and lamotrigine 200 mg/day. She had no obvious physical or neurological abnormalities. A 12-lead electrocardiogram (ECG) showed no arrhythmia. No abnormalities were seen on a blood exam, and no organic abnormalities were present on head magnetic resonance imaging. The seizures were symmetrical generalized tonic-clonic seizures (GTCS) without aura, and the patient was unable to stand up for several hours after the seizure. In addition, she had a "sandstorm" in front of her eyes, chest discomfort, cold sweating, and LOC, and we suspected mixed syncope. To confirm the diagnosis and determine the future treatment plan, long-term video electroencephalogram (EEG) monitoring was performed. The background was a normal posterior dominant rhythm of 9-10 Hz, and the interictal findings were generalized spikes and wave complexes (Figure 1A). Two types of seizure findings were obtained: (1) GTCS starting with generalized polyspikes and waves (Figure 1B) and (2) syncope with sinus arrest for up to 10 seconds when the patient tried to stand up after GTCS (Figure 1C).

As a result, the seizure type was determined to be GTCS, the epilepsy type was considered generalized epilepsy, and a co-morbidity of syncope was diagnosed. After the addition of valproic acid 600 mg/day following the diagnosis of generalized epilepsy, the epileptic seizures disappeared, but syncope remained. We consulted the cardiology department of our hospital and diagnosed mixed neuromodulatory syncope after performing the tilt test. She underwent catheter ablation for cardioneuromodulation, and her syncope improved.

## Discussion

This is the first report of a case in which both epileptic seizures and neuromodulatory syncope were captured by video EEG monitoring and required specialized treatment for each. In epilepsy-related syncope, ictal bradycardia or ictal syncope is well known and has been implicated in sudden unexpected death in epilepsy (SUDEP) [6,7]. However, in this case, ictal asystole or bradycardia was not observed, and asystole and syncope were induced during the interictal period, suggesting the existence of another pathology. The fact that repositioning induced syncope after the seizure suggested that autonomic nervous system abnormalities, mainly decreased baroreflex sensitivity (BRS), may be involved in the pathogenesis of this case.

After an epileptic seizure, an increase in blood pressure and tachycardia due to sympathetic nervous system excitation is typical, but bradycardia, shallow breathing, and apnea may also occur [8]. Recently, the blood pressure changes associated with focal seizures have been reported to be moderate, and BRS does not change significantly [9,10]. On the other hand, in bilateral convulsive seizure (BCS), BRS appears to decrease significantly after the seizure, and post-ictal blood pressure appears to drop rapidly to baseline levels or lower [11,12]. Post-paroxysmal bradycardia and hypotension are facilitated by skeletal muscle metabolism-mediated muscle hyperemia and decreased BRS after BCS, resulting in inadequate blood supply to various tissues and, in some cases, fatal hypoperfusion of body organs, which may lead to SUDEP [7,13]. These reports suggest that a similar phenomenon may occur in GTCS and may be the cause of the syncope in the present case.

In addition to suppression of epileptic seizures, when autonomic symptoms associated with epilepsy are severe, as in this case, careful cardiovascular examination and treatment are essential with the goal of preventing SUDEP. Regardless of the duration of syncope, patients with syncope should be considered for a cardiac pacemaker. Its usefulness is not yet clear, but it may be effective in cases of fatal arrhythmias [14,15]. However, because a pacemaker does not fundamentally resolve BRS, it may improve arrhythmias but not hypotension due to vasodilation. Vagal nerve stimulation may improve autonomic neuropathy and prevent SUDEP due to its feedback to the central nervous system and cardioprotective effects, but its efficacy is unknown [16]. In recent years, a number of reports have described the treatment of neuromodulatory syncope with cardiac catheter ablation [17,18]. Scattered reports of improvement in BRS following cardiac catheter ablation have also been published.

## Conclusions

This case report highlights the importance of considering both epilepsy and syncope in the differential diagnosis of patients with recurrent transient loss of consciousness. Baroreflex sensitivity (BRS) related to epileptic seizures may lead to SUDEP. When autonomic symptoms of epilepsy are severe, as they were in this case, treatment should focus on avoiding SUDEP in addition to suppressing epileptic episodes. A comprehensive cardiovascular examination is also important. In young people such as in the present case, cardiac catheter ablation or implantation of a cardiac pacemaker should be considered.
